# Natural Aging of a Local Indonesian Rice Variety: Physical, Chemical, and Cooking Quality Analyses

**DOI:** 10.1155/ijfo/6610135

**Published:** 2025-12-18

**Authors:** Purwa Tri Cahyana, Titi Candra Sunarti, Erliza Noor, Hartrisari Hardjomidjojo, Noer Laily

**Affiliations:** ^1^ Department of Agroindustrial Technology, Faculty of Agricultural Engineering and Technology, IPB University, Bogor, Indonesia, ipb.ac.id; ^2^ Research Center for Agroindustry, National Research and Innovation Agency, KST BJ Habibie, South Tangerang, Indonesia, brin.go.id; ^3^ Research Center for Food Technology and Processing, National Research and Innovation Agency, Yogyakarta, Indonesia, brin.go.id

**Keywords:** cooking properties, natural aging, physicochemical, rice, texture

## Abstract

Rice (*Oryza sativa*) of the Ciherang variety has been widely stored in warehouses for years as government food reserves. Storing rice for a long time causes a decrease in quality due to the natural aging process. This study is aimed at revealing the quality parameters most affected by the natural aging of rice during long‐term storage (12–48 months). Changes in rice physical properties (including texture), chemical properties, and cooking quality were analyzed at 0, 12, 24, 36, and 48 months of storage, and Pearson correlation coefficients were calculated to determine the relationships between the parameters. Bulk density varied from 0.88 ± 0.00 to 0.79 ± 0.17 g/mL, insect excreta (frass), that is, secretions and metabolic residues from storage pests start from 0.00*%* ± 0.00*%* to 3.02*%* ± 0.70*%*, total dissolved solids from 245.33 ± 1.53 to 508.00 ± 83.07 mg/L, yellowness (*b*∗) from 12.13 ± 0.07 to 15.87 ± 0.19, free fatty acids from 0.26*%* ± 0.01*%* to 0.76*%* ± 0.06*%*, hardness from 7.73 ± 0.97 to 12.49 ± 2.09 kgf, cooking time from 23.40 ± 0.58 to 25.40 ± 0.44 min, and water uptake ratio from 3.70 ± 0.03 to 4.32 ± 0.34 g/g. Yellowness and total dissolved solids were significantly affected by storage time, exhibiting a strong positive correlation (*r* = 0.930, *p* < 0.01; *r* = 0.908, *p* < 0.01, respectively). In contrast, the water uptake ratio and free fatty acids were less influenced by storage time. The presence of foreign matter was strongly and positively correlated with both yellowness and the total dissolved solids. Mitigating yellowness and degradation of the grains is very important to maintain the quality of Ciherang rice during extended storage duration.

## 1. Introduction

Rice (*Oryza sativa* L.) is among the most important staple foods globally, especially in Asia, and is a source of nutrition for billions of consumers [1]. Indonesian rice is economically and culturally important and is a key crop in national food security strategies. The Indonesian government, striving to ensure supply, warehouses rice, particularly the Ciherang variety, as a long‐term food inventory. Ciherang is extensively cultivated because it possesses qualities that are favorable to farmers, such as producing high yields, being resistant to significant diseases, and having excellent grain quality [2]. Storage for an extended period of time under ambient tropical temperatures, nevertheless, leads to progressive loss of quality in rice due to natural aging, which changes its physical, chemical, and cooking characteristics [3, 4]. The natural aging process generally takes a minimum of 4 months [5].

Natural senescence of rice during storage causes several physicochemical and sensory changes. These include increased hardness [6, 7], yellowness [8], and free fatty acid (FFA) content [9–13], and reduced bulk density [14] and water absorption [13, 15, 16]. More seriously, long‐term storage promotes the growth of insect frass, indicating biological deterioration and infestation. Insect frass, a fine dust composed of insect feces and waste grain, has been a widely reported marker of postharvest loss and pest infestation, such as *Sitophilus oryzae* (L.), in tropical storage environments [17, 18].

Changes in key indicators, chromatic parameters (notably *b*∗), were found to correlate significantly with storage duration. These changes not only reduce the esthetic appeal of the grain but may also affect its nutritional and functional properties [19, 20]. Moreover, starch degradation and lipid oxidation further compromise the cooking quality and consumer acceptability [21]. Despite the relevance of this topic, few studies have comprehensively assessed the multidimensional deterioration of rice stored under real‐world conditions in tropical developing countries.

This study was conducted to identify the rice quality traits that are most affected by natural aging during long‐term storage (up to 48 months) of Ciherang rice in government warehouses. This study, based on physicochemical properties, cooking quality, and biological deterioration indicators such as frass formation by insects, provides valuable information for the management of storage systems and rice quality in tropical climates.

## 2. Materials and Methods

### 2.1. Materials

**Figure 1:**
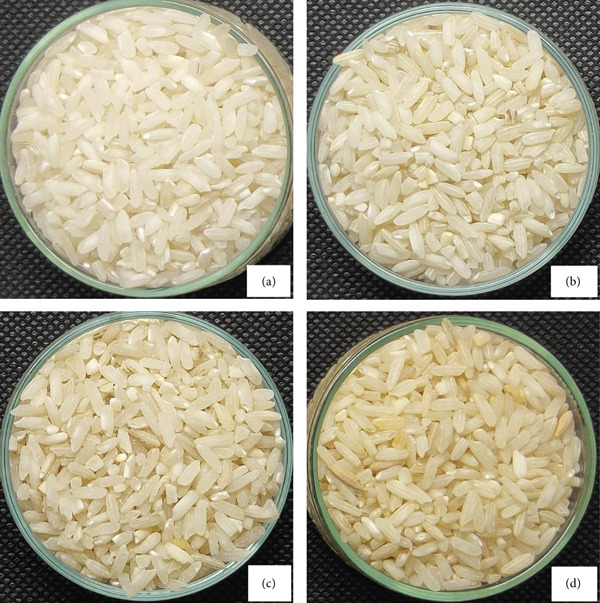
Photograph of the appearance of milled rice (Ciherang variety) after different storage times: (a) 12, (b) 24, (c) 36, and (d) 48 months.

Milled rice of local Indonesian rice (Ciherang variety) from the Bulog Warehouse, West Java Regional Division in Indonesia, was harvested and milled in June 2019 prior to storage. Freshly milled rice (0 months of storage) was analyzed immediately after milling to serve as a baseline sample, hereafter referred to as “fresh rice.” The initial moisture content of the fresh rice was 11.69*%* ± 0.06*%*, the amylose content was 24.39*%* ± 0.99*%*, and the length‐to‐width ratio of the rice grains was 2.82 ± 0.03. Following baseline analysis, rice samples were stored for 12, 24, 36, and 48 months (Figure 1) under ambient conditions in the Bulog Warehouse at 31.3^°^C ± 0.9^°^C and 58*%* ± 6.5*%* relative humidity. The 12‐month storage period was calculated from June to June of the following year, with similar calculations for the 24‐, 36‐, and 48‐month storage periods, respectively.

### 2.2. Bulk Density Measurement

Bulk density measurements were performed following the method described by Singh et al. [22]. Rice samples were added to a measuring cup until they reached a specific volume, and the bulk density was reported in grams per milliliter.

### 2.3. Frass Measurements

The mass of particles traversing an 18 mesh sieve (sieve aperture diameter of 1 mm) was quantified. A 100 g rice sample was sieved using an 18 mesh sieve (Retsch Test Sieve). The weight of the frass was determined relative to the dry matter as follows:

Frass %=weight of frassweight of sample×100%.

### 2.4. Total Dissolved Solid (TDS) Analysis

The TDSs are measured using a TDS meter, according to Thupeeban and Kannan′s method [23]. The rice was soaked in distilled water at a 1:2 (rice to distilled water) ratio for 120 min. After soaking, the solution was left undisturbed for 30 min to allow the suspended particles to settle, and the TDS was measured.

### 2.5. FFA Content

FFA was tested using the method described by Lu et al. [24]. Rice flour was extracted using anhydrous ethanol at room temperature. The filtered solution was added to phenolphthalein reagent, titrated with 0.01 N KOH standard solution, and the FFA content was calculated using the following equation:

FFA %=V KOH×N KOH×MW weight of sample ×100%

where *V* is the volume of titrant, *N* is the normality of titrant, and MW is the molecular weight of fatty acids.

### 2.6. Measurement of Grains′ Hardness

The hardness of the head rice grains was measured according to the method described by Tao et al. [16] with modifications. Hardness measurements were performed on intact rice grains placed at the center of the heavy‐duty platform of a TA.XT Plus Texture Analyzer (Stable Micro Systems, United Kingdom) and pressed with a 5 mm diameter P/5 probe. The testing settings were as follows: pretest speed, 0.50 mm/s; test speed, 0.50 mm/s; posttest speed, 10.00 mm/s; strain, 40%; and trigger force, 5.0 N.

### 2.7. Grains′ Color Evaluation

Color analysis was performed using the method outlined by Liu et al. [25]. The color change of the rice grains was measured using a colorimeter (CR‐410, Konica Minolta, Japan) with D65 illumination. The parameters *L*∗, *a*∗, and *b*∗ are used to characterize chroma: *L*∗ represents the spectrum of light from darkness (0) to light (100), *a*∗ represents the spectrum of green (−*a*∗) to red (+*a*∗), and *b*∗ measures the yellow spectrum from blue (−*b*∗) to yellow (+*b*∗).

### 2.8. Determination of Cooking Time (CT)

The method described by Bhat and Riar [26] was used to determine CT. A 2 g sample of head rice was cooked in a test tube containing 20 mL of distilled water and placed in a boiling water bath. The CT was measured at various intervals by pressing the rice grains between two glass plates until no white core remained in the rice grains.

### 2.9. Determination of Water Uptake Ratio (WUR)

The WUR was measured following the method described by Bhat and Riar [26]. Two grams of head rice was added to 20 mL of distilled water in a test tube and boiled until cooked. After cooking, the remaining water was separated from the cooked rice, the rice was weighed, and the WUR was calculated in grams per gram (g/g).

Water uptake ratio=weight of cooked rice weight of uncooked rice .

### 2.10. Surface Morphological Analysis

Observation of the surface morphology of rice grains refers to the method of Chen et al. [27] using a scanning electron microscope (SEM) (JEOL JSM, IT200, Japan). The surface morphology of rice grains was observed at various levels of damage caused by pests in warehouses. The samples were inserted into a sample holder, sputter‐coated with gold, and then inserted into the specimen chamber of the SEM. Photography was performed at a magnification of 40×. The photographed samples were grains with varying levels of damage based on weight loss, including (a) whole grain rice, (b) grains with 25% weight loss, (c) grains with 50% weight loss, and (d) grains with 75% weight loss.

### 2.11. Statistical Analysis

The results are presented as mean values with standard deviations. All tests were conducted in triplicate, except for color evaluation and grain hardness, which were performed 10 times. The acquired data were processed by analysis of variance (ANOVA) using SPSS Statistics, Version 26.0 (IBM Corp., New York, United States). Differences were considered significant at *p* ≤ 0.05. Tukey′s HSD test was applied when the assumption of homogeneity of variances was met, whereas the Games–Howell test was used when this assumption was violated. The Pearson correlation coefficient (*r*) was used to examine the interrelationship between storage time and the physical, chemical, and cooking properties. Detailed outputs of all statistical analyses are provided in the supporting information.

## 3. Result and Discussion

### 3.1. Bulk Density

**Figure 2:**
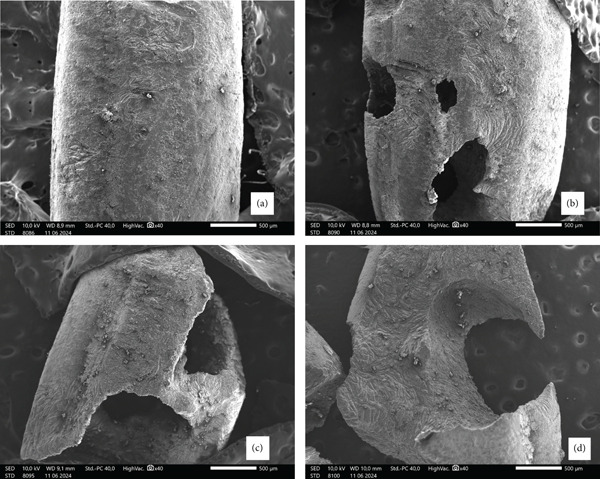
SEM analysis of the surface of rice grains (Ciherang variety) at 40× magnification: (a) undamaged rice grain, (b) 25% weight loss, (c) 50% weight loss, and (d) 75% weight loss.

According to Utami et al. [28], the bulk density of local Indonesian rice ranges from 0.79 to 0.89 g/mL. The bulk density of milled rice (Ciherang variety) from different storage times is shown in Table 1. The bulk density of rice with a storage period of 0 months (fresh rice) was 0.88 + 0.00 g/mL, whereas that of rice with a storage period of 48 months was 0.79 + 0.17 g/mL. This indicates that the bulk density decreased as the storage period increased. When comparing the storage period of 48 months with that of 12 months, the bulk density decreased by ±9.2%. The bulk density of rice stored for 48 months was significantly different from that stored for other periods (*p* < 0.05). The decrease in bulk density for more extended storage periods indicates that the weight of the rice has decreased, possibly caused by the loss of some of the endosperm in the rice grains [14]. According to Doherty et al. [18], considerable physical damage to rice is caused by the rice weevil *S. oryzae* (L.), which causes weight loss due to the porous part of the grain being eaten by this pest. Ndomou et al. [29] also stated that the rice weevil (*S. oryzae*) is one of the most harmful pests affecting whole grains. Figure 2 shows the SEM analysis of the surface of rice grains (Ciherang variety) damaged by the beetle activity. The observed grain damage caused by insects (Figure 2) was managed through ongoing pest mitigation measures implemented throughout the storage period. Routine phosphine fumigation and residual spraying reduced infestation pressure in line with best practice protocols for tropical rice storage [30].

**Table 1:** Physicochemical properties of rice (Ciherang variety) with variations in storage time.

**Physicochemical properties**	**Storage time (months)**				
	**0**	**12**	**24**	**36**	**48**
Bulk density (g/mL)	0.88 ± 0.00^a^	0.87 ± 0.01^a^	0.86 ± 0.01^a^	0.85 ± 0.02^a,b^	0.79 ± 0.17^b^
Frass (%)	0.00 ± 0.00^a^	0.01 ± 0.00^a^	0.03 ± 0.02^a^	0.19 ± 0.17^a^	3.02 ± 0.70^b^
Total dissolved solids (mg/L)	245.33 ± 1.53^a^	264.67 ± 48.58^a^	293.67 ± 27.57^a^	402.33 ± 58.77^a^	508.00 ± 83.07^a^
Free fatty acids (%)	0.26 ± 0.01^a^	0.60 ± 0.06^b^	0.63 ± 0.15^b^	0.67 ± 0.17^b,c^	0.76 ± 0.06^c^
Hardness (kgf)	7.73 ± 0.97^a^	10.71 ± 2.84^b^	11.32 ± 2.50^b^	11.89 ± 2.87^b^	12.49 ± 2.09^b^

*Note:* Values with the same letter in the same row are not significantly different at *p* < 0.05 (Games–Howell test).

The Pearson correlation coefficient (*r*) displaying the relationship between storage time and physical properties, chemical properties, and cooking quality of rice, as well as between other parameters, is shown in Table 2. According to Schober and Schwarte [31], a variable has a very strong correlation if it has a correlation coefficient ≥ 0.90 and a strong correlation if it has a correlation coefficient 0.70–0.89. Table 2 shows that bulk density was strongly and negatively correlated with storage time (*r* = −0.887, *p* < 0.01). Bulk density also had a very strong negative correlation with yellowness (*r* = −0.932, *p* < 0.01) and frass content (*r* = −0.939, *p* < 0.01).

**Table 2:** Pearson analysis of the physicochemical, texture, and cooking quality (Ciherang variety) with variations in storage time.

	**BD (g/mL)**	**Frass (%)**	**Yellowness (** **b**∗**)**	**TDS (mg/L)**	**FFA (%)**	**CT (min)**	**WUR (g/g)**	**Hardness (kgf)**
Frass	−0.939 ^∗∗^							
Yellowness	−0.932 ^∗∗^	0.882 ^∗∗^						
TDS	−0.836 ^∗∗^	0.758 ^∗∗^	0.838 ^∗∗^					
FFA	−0.533 ^∗^	0.392	0.561 ^∗^	0.487 ^∗^				
CT	−0.608 ^∗∗^	0.548 ^∗^	0.707 ^∗∗^	0.616 ^∗∗^	0.569 ^∗∗^			
WUR	−0.631 ^∗∗^	0.494 ^∗^	0.556 ^∗^	0.460 ^∗^	0.433	0.567 ^∗∗^		
Hardness	−0.720 ^∗∗^	0.612 ^∗∗^	0.818 ^∗∗^	0.776 ^∗∗^	0.709 ^∗∗^	0.785 ^∗∗^	0.582 ^∗∗^	
Storage time	−0.887 ^∗∗^	0.794 ^∗∗^	0.930 ^∗∗^	0.908 ^∗∗^	0.608 ^∗∗^	0.825 ^∗∗^	0.581 ^∗∗^	0.881 ^∗∗^

Abbreviations: BD, bulk density; CT, cooking time; FFA, free fatty acid; TDS, total dissolved solid; WUR, water uptake ratio.

^**^Correlation is significant at the 0.01 level;  ^∗^significant at the 0.05 level.

### 3.2. Insect Excreta (Frass)

The frass content increased significantly from nearly zero (0.00*%* ± 0.00*%*) at 0 months of storage to 3.02*%* ± 0.70*%* at 48 months (Table 1). The frass detected consisted of powder from the screening that passed through Mesh 18. Frass content can serve as an indicator of rice damage. With more extended storage periods, there is a potential for increased frass owing to the deterioration of rice. Rice stored for 48 months had the highest amount of frass and significantly differed from other storage times (*p* < 0.05). Rice stored for 48 months showed significant deterioration. Singh et al. [32] observed that rice beetles often damage rice grains, leading to hollow grains, disintegration, and powder formation. In rice, impurities are generally more abundant when *S. oryzae* is present, causing a decrease in grain weight [33]. Figure 2 shows the damage to rice grains caused by weevil attacks. The frass is an indicator of rice deterioration [34]. Adult beetles feed on the endosperm, whereas larvae consume the seeds inside the grains [35]. Table 2 shows that frass was strongly correlated with storage time (*r* = 0.794, *p* < 0.01). Additionally, frass had a very strong negative correlation with bulk density (*r* = −0.939, *p* < 0.01). The lower the bulk density, the higher the likelihood of rice deterioration, as indicated by the presence of frass.

### 3.3. TDSs

Table 1 shows that the TDS increased from 245.33 ± 1.53 *m* *g*/*L* (0 months of storage) to 508.00 ± 83.07 *m* *g*/*L* (48 months of storage). Although ANOVA indicated overall significant differences (*p* < 0.001), the Games–Howell post hoc test did not reveal significant pairwise differences, likely due to the high within‐group variability and stricter multiple‐comparison adjustments. According to Table 2, TDS exhibited a strong positive correlation with the storage period (*r* = 0.908, *p* < 0.01). Therefore, TDS also tends to increase with storage period. Additionally, TDS showed a strong positive correlation with foreign matter (*r* = 0.758, *p* < 0.01). Consequently, rice stored for 48 months contained more dissolved particles. Zhu et al. [36] stated that during soaking, solids composed of starch, protein, and small‐molecule substances leach from the surface and cracks of the rice grains into the soaking water. According to Gong et al. [37], physical damage to grains causes the endosperm layer to be rapidly eroded by water, resulting in more dissolved solid particles. Soaking also causes some rice starch, protein, and minerals to dissolve [38].

### 3.4. FFA Content

Table 1 shows the FFA content of rice flour (Ciherang variety) stored for 0, 12, 24, 36, and 48 months at room temperature. The FFA of rice flour varied from 0.26*%* ± 0.01*%* to 0.76*%* ± 0.06*%*. Compared to 12 and 48 months of storage, the FFA increased by 26% and revealed significant differences (*p* < 0.05). As rice ages naturally, some of its fat undergoes hydrolysis into FFA, which oxidizes and decomposes, resulting in an unpleasant taste [11]. Therefore, the FFA content in rice can indicate a decrease in rice quality during storage. Cai et al. [39] reported that when brown rice is stored at 30^°^C ± 1^°^C with a relative humidity of 60*%* ± 5*%*, lipids undergo decomposition through the action of esterases, resulting in the production of FFAs. Lipids change most significantly during rice storage, followed by starch, whereas proteins change slowly [24]. Indica rice (Taiyou 217) stored at 25°C and 75% relative humidity for 12 months has also been shown to reduce fat and protein content by 47% and 4%, respectively. However, for japonica rice, the fat and protein contents decreased by 37% and 5%, respectively [21]. Although rice contains a relatively small amount of fat, this component significantly influences quality changes during storage. Lipids in rice grains are susceptible to free radical attack, leading to oxidation and peroxidation. Elevated temperatures and increased oxygen concentrations can accelerate lipid rancidity, resulting in the deterioration of the quality of the stored rice [40]. Additionally, lipid hydrolysis results in the formation of numerous FFAs, which emit an unpleasant odor over time [41, 42]. FFA readily interacts with oxygen, leading to the formation of lipid peroxides and other oxidative products, such as volatile short‐chain aldehydes and ketones, which contribute to off‐flavors and reduced shelf life [43, 44]. Shen et al. [3] observed that increased lipase activity during rice storage resulted in higher FFA content. FFAs were formed more rapidly at higher temperatures [45]. During a 12‐month storage period at 37°C and 68% humidity, the aroma quality of glutinous rice deteriorated more rapidly than that of japonica or indica rice. This decline is attributed to the reduction in soluble sugar content and the occurrence of the Maillard reaction during cooking [10]. From Table 2, Pearson analysis exhibited a moderate positive correlation (*r* = 0.608, *p* < 0.01) between the FFA and storage time.

### 3.5. Grains′ Hardness

Table 1 shows that the hardness ranged from 7.73 ± 0.97 to 12.49 ± 2.09 *k* *g* *f*. The hardness of rice stored for 48 months was the highest but was not significantly different from that stored for 12, 24, and 36 months (*p* < 0.05). The hardness of rice increased with storage time of 12, 24, and 36 months but was not significantly different. The increased hardness may have resulted from structural changes in the main constituents of rice, including starch, lipids, and proteins [6]. Prior studies have demonstrated that the natural aging process of rice leads to increased stiffness and strength of its cell walls, attributed to protein cross‐linking [45]. Research conducted on Indonesian rice varieties, specifically Mentik Wangi, revealed that after being stored for 6 months (27°C–30°C and 54%–62% humidity), the hardness also increased from 0.71 ± 0.42 to 2.39 ± 0.81 *N* [46]. Table 2 indicates that hardness was strongly correlated with storage time (*r* = 0.881, *p* < 0.01).

### 3.6. Grains′ Color Evaluation

As shown in Table 3, rice stored for 0 months had the highest brightness (*L*∗), which did not differ with 12 months of storage time but was significantly different from that stored for 24, 36, and 48 months. Meanwhile, the redness (*a*∗) of rice was significantly different after 48 months of storage. The highest yellowness (*b*∗) was found in rice stored for 48 months (15.87 ± 0.19), which was significantly different from that of rice stored for other periods. Yellowing of rice is a sign of decreasing quality and value of the rice commodity, indicated by the *b*∗ value [19]. Figure 1 shows that rice stored for 48 months appeared the most yellow compared to rice stored for shorter periods.

**Table 3:** Cooking properties of rice (Ciherang variety) with variations in storage time.

**Cooking properties**	**Storage time (months)**				
	**0**	**12**	**24**	**36**	**48**
Cooking time (min)	23.40 ± 0.58^a^	23.70 ± 0.79^a^	24.60 ± 0.61^b^	25.00 ± 0.74^b,c^	25.40 ± 0.44^c^
Water uptake ratio (g/g)	3.70 ± 0.03^a^	3.76 ± 0.29^a^	3.98 ± 0.34^b^	4.06 ± 0.48^b,c^	4.32 ± 0.34^c^

*Note:* Values with the same letter in the same row are not significantly different at *p* < 0.05 (Tukey HSD test).

Several studies have revealed that the increase in yellow color in rice due to the aging process is caused by the Maillard reaction and heat stress [19, 47], moisture content [25], storage duration, and storage temperature [48]. According to Wang et al. [49] and Mohammadi Shad et al. [50], the carbonyl groups in starch‐based reducing sugars and the amino groups in rice proteins undergo a non‐enzymatic Maillard reaction, resulting in rice yellowing.. Similarly, Liu et al. [51] showed that secondary metabolites, such as flavonoids and phenylpropanoids, were significantly accumulated in yellowed rice and that hydroxymethylfuraldehyde (HMF) was produced as a result of the Maillard reaction. However, Liu et al. [19] discovered that the yellowing of rice during storage is a multifaceted process, with the Maillard reaction being a potential mechanism. They suggested that heat stress is the main trigger for rice yellowing, as evidenced by the increased levels of heat shock proteins, late embryogenesis abundant proteins, and proteins related to desiccation. Yellowness showed a very strong positive correlation (*r* = 0.930, *p* < 0.01) with storage time (Table 2).

### 3.7. CT

The CT for rice stored for different storage times ranged from 23.40 ± 0.58 to 25.40 ± 0.44 min (Table 4). Rice stored for 48 months exhibited the longest CT, which was significantly different from that of rice stored for 12 and 24 months (*p* < 0.05). However, there was no significant difference in the CT between the 48‐ and 36‐month storage periods. A longer CT indicates slower water penetration into the rice grains, resulting in an extended CT. Zhou et al. [52] stated that the aging process causes gelatinization to take longer than that in fresh rice. Ziegler et al. [53] also revealed that storing brown rice at 32°C for 6 months caused the CT to increase from 23 to 25.6 min. Table 2 shows that CT was strongly correlated with storage time (*r* = 0.825, *p* < 0.01).

**Table 4:** Color value of rice (Ciherang variety) grains with variations in storage time.

**Color value**	**Storage time (months)**				
	**0**	**12**	**24**	**36**	**48**
Lightness (*L*∗)	76.27 ± 0.19^a^	76.12 ± 0.19^a^	73.68 ± 0.51^b^	73.66 ± 0.43^b^	73.57 ± 0.08^b^
Redness (*a*∗)	1.68 ± 0.16^a^	1.81 ± 0.20^a^	1.98 ± 0.39^a,b^	2.23 ± 0.17^b^	2.94 ± 0.06^c^
Yellowness (*b*∗)	12.13 ± 0.07^a^	12.18 ± 0.13^a^	13.40 ± 0.42^b^	13.72 ± 1.07^b^	15.87 ± 0.19^c^

*Note:* Values with the same letter in the same row are not significantly different at *p* < 0.05 (Games–Howell test).

### 3.8. WUR

Table 3 shows the WUR of milled rice (Ciherang variety) stored for 0, 12, 24, 36, and 48 months. The WUR of rice ranged from 3.70 ± 0.03 to 4.32 ± 0.34 *g*/*g* and increased with storage time. Rice storage for 0 and 12 months did not result in significant differences in the WUR. However, they showed significant differences between storage times of 12 and 48 months (*p* < 0.05). The WUR was affected by storage time [6]. Other studies have shown that the WUR is influenced by amylose content (Suismono et al. 2022). The increased WUR can also be attributed to amylase activity and storage at moderate temperatures [54]. Hadipernata et al. [55] also revealed that milled rice stored at room temperature for 8 months experienced an increase in WUR. Pearson analysis showed that the WUR had a moderate positive correlation with storage time (*r* = 0.581, *p* < 0.01). In this study, the WUR had a moderate negative correlation with bulk density (*r* = −0.631, *p* < 0.01).

According to Pearson′s correlation analysis (Table 2), the parameters most significantly affected by natural aging are yellowness (*r* = 0.930, *p* < 0.01) and TDSs (*r* = 0.908, *p* < 0.01). Both factors demonstrated a very strong positive correlation with storage time. TDS showed a strong positive correlation with frass (*r* = 0.758, *p* < 0.01) and a negative correlation with bulk density (*r* = −0.836, *p* < 0.01). Based on the parameter testing in this study, significant changes in the quality of Ciherang rice occurred after 36 months of storage. To slow down the damage to Ciherang rice, it is necessary to note the factors that accelerate the yellowing process, including temperature and humidity. The higher the temperature of the warehouse and the humidity, the faster the yellowing process. Preventing damage to rice grains due to rice beetle attacks is also an important factor that needs to be considered.

### 3.9. Surface Morphological Analysis

In addition to the intrinsic effects of natural aging on rice quality, prolonged storage increases the risk of insect infestation, which represents a distinct mechanism of deterioration. Insect‐induced grain damage compromises the integrity of rice kernels by creating perforations and frass deposits, which accelerate water absorption and solid leaching during cooking. Therefore, insect‐related deterioration should be considered separately from natural aging effects as it introduces an additional pathway for quality loss during extended storage. Figure 2 shows an analysis of the surface morphology of rice grains (Ciherang variety) at several levels of damage due to weevil activity. The average weight of each grain was 0.022 ± 0.002 *g*. Rice grains that are not affected by weevils appear intact. However, rice grains weighing 0.017 g showed a 25% weight loss and exhibited signs of beetle damage. A grain weighing 0.008 g experienced a 50% weight loss and had larger holes, indicating extensive damage. In comparison, a grain weighing 0.002 g underwent a 75% weight loss and showed signs of significant beetle consumption, such as cut grains. The presence of damaged or porous grains leads to a decrease in both grain weight and bulk density. Over time, longer storage periods result in increased damage to rice grains, as both larvae and adult beetles feed on the interior of the grains. The bulk density (Table 1) ranged from 0.87 ± 0.01 to 0.79 ± 0.17 *g*/*m* *L*, indicating that beetle attacks decrease rice grain density, particularly as the storage period extends. Adult insects typically feed on the endosperm beneath the husk, while larvae consume the germ inside the grain [56]. Overall, insect infestation during storage accelerates quality deterioration beyond the biochemical changes driven by natural aging. The loss of kernel mass, reduction in bulk density, and presence of frass not only lower the commercial and nutritional value of rice but also increase susceptibility to microbial contamination and further degradation. These effects highlight the importance of implementing effective pest management strategies, such as hermetic storage, controlled atmospheres, or insect‐resistant packaging materials, to preserve grain integrity during extended storage. By separating pest‐induced deterioration from the intrinsic effects of natural aging, this study emphasizes the need to consider both mechanisms when evaluating long‐term rice storage outcomes.

Custodio et al. [57] reported that consumer perceptions of rice quality differ across regions: While Southeast Asian consumers emphasize aroma and texture, South Asian consumers prioritize whiteness and uniformity of grains. However, in both regions, discoloration or yellowish grains were consistently associated with poor‐quality rice. Although the yellowness (*b*∗) of rice increases progressively during storage and is widely recognized as a key indicator of quality deterioration, no published quantitative threshold defines the maximum *b*∗ value at which rice is still considered edible. Most studies report only relative increases over time [19, 20, 47, 51, 58], while reviews emphasize that yellowish grains are consistently perceived by consumers and industry as low‐quality rice [57].

According to Wang et al. [59], rice with an FFA value below 0.81% is considered edible. Since the FFA content of Ciherang rice in this study was 0.67% after 36 months of storage, it still met the criteria for edible rice. In this study, Ciherang rice stored at room temperature for up to 36 months showed FFA levels below 0.8%. Qiu et al. [60] revealed that Yumepirika rice had 0.65% FFA after being stored for 38 months at room temperature. After 36 months, rice shows more yellowing and chemical changes, making it poor quality by consumers and the rice industry [57]. This suggests that storing rice for up to 36 months is the maximum limit for maintaining acceptable quality.

Custodio et al. [57] also stated that one of the criteria for acceptable rice quality is that uncooked grains should have minimal impurity. In our study, frass levels were 0.03% after 24 months and 0.19% after 36 months of storage (Table 1), values that can still be considered negligible and consistent with the definition of minimal impurities. However, after 48 months, frass increased sharply to 3.02%, representing visible contamination that would reduce consumer acceptability of the product. These results indicate that rice stored for up to 36 months can still be categorized as edible with acceptable impurity levels, whereas storage beyond this duration results in excessive insect damage and a transition to poor quality.

## 4. Conclusions

This study demonstrated that prolonged storage of Ciherang rice under ambient conditions (31.3^°^C ± 0.9^°^C, 58*%* ± 6.5*%* RH) led to significant changes in physical, chemical, and cooking quality parameters. The results indicated that storage duration had the most significant impact on the yellowness and TDSs. Furthermore, the TDS was notably influenced by frass and the bulk density. The results of this study indicate that the Ciherang variety of rice should not be stored under these environmental conditions for more than 36 months. This finding highlights the necessity of inhibiting or preventing yellowing and physical damage to Ciherang rice stored for extended periods at ambient temperatures. This is essential for maintaining the quality of rice. To mitigate these effects, practical measures such as hermetic or modified atmosphere packaging, prestorage drying to safe moisture levels, temperature moderation, and integrated pest management are recommended to preserve rice quality and consumer acceptability, respectively.

## Conflicts of Interest

The authors declare no conflicts of interest.

## Funding

No funding was received for this manuscript.

## Supporting information


**Supporting Information** Additional supporting information can be found online in the Supporting Information section. The supporting information includes detailed statistical analyses supporting the data presented in this manuscript. The files are as follows: statistical analysis (bulk density), statistical analysis (CT), statistical analysis (FFA), statistical analysis (frass), statistical analysis (grains′ color), statistical analysis (hardness), statistical analysis (TDS), and statistical analysis (WUR). Each file provides the complete results of the statistical tests, including the mean values, standard deviations, and significance levels for each measured parameter. This supporting information supports the transparency and reproducibility of the analyses performed in this study.

## Data Availability

All the data used in this study is included in the manuscript itself.
