# Supplementary material for: Natural Aging of a Local Indonesian Rice Variety: Physical, Chemical, and Cooking Quality Analyses
Source: Int J Food Sci. 2025 Dec 18;2025:6610135. doi: 10.1155/ijfo/6610135 (PMC12714122; doi:10.1155/ijfo/6610135)
Supplement: Supplementary file 1 — Supporting Information Additional supporting information can be found online in the Supporting Information section. The supporting information includes detailed statistical analyses supporting the data presented in this manuscript. The files are as follows: statistical analysis (bulk density), statistical analysis (CT), statistical analysis (FFA), statistical analysis (frass), statistical analysis (grains′ color), statistical analysis (hardness), statistical analysis (TDS), and statistical analysis (WUR). Each file provides the complete results of the statistical tests, including the mean values, standard deviations, and significance levels for each measured parameter. This supporting information supports the transparency and reproducibility of the analyses performed in this study. [file IJFO-2025-6610135-s001.zip › Statistical analysis (Frass).docx]

**Statistical analysis (Frass).**

| **Tests of Normality** | | | | | | | |
| --- | --- | --- | --- | --- | --- | --- | --- |
|  | Storage time | Kolmogorov-Smirnov^a^ | | | Shapiro-Wilk | | |
|  |  | Statistic | df | Sig. | Statistic | df | Sig. |
| Frass | 0 month | . | 3 | . | . | 3 | . |
|  | 12 months | .292 | 3 | . | .923 | 3 | .463 |
|  | 24 months | .362 | 3 | . | .803 | 3 | .122 |
|  | 36 months | .252 | 3 | . | .965 | 3 | .643 |
|  | 48 months | .176 | 3 | . | 1.000 | 3 | .975 |
| a. Lilliefors Significance Correction | | | | | | | |

| **One-Sample Kolmogorov-Smirnov Test** | | | |
| --- | --- | --- | --- |
|  | | | Unstandardized Residual |
| N | | | 15 |
| Normal Parameters^a,b^ | Mean | | .0000000 |
|  | Std. Deviation | | 1.00233364 |
| Most Extreme Differences | Absolute | | .164 |
|  | Positive | | .139 |
|  | Negative | | -.164 |
| Test Statistic | | | .164 |
| Asymp. Sig. (2-tailed) | | | .200^c,d^ |
| Monte Carlo Sig. (2-tailed) | Sig. | | .752^e^ |
|  | 99% Confidence Interval | Lower Bound | .741 |
|  |  | Upper Bound | .763 |
| a. Test distribution is Normal. | | | |
| b. Calculated from data. | | | |
| c. Lilliefors Significance Correction. | | | |
| d. This is a lower bound of the true significance. | | | |
| e. Based on 10000 sampled tables with starting seed 2000000. | | | |

| **Test of Homogeneity of Variances** | | | | | |
| --- | --- | --- | --- | --- | --- |
|  | | Levene Statistic | df1 | df2 | Sig. |
| Frass | Based on Mean | 3.631 | 4 | 10 | .045 |
|  | Based on Median | 3.239 | 4 | 10 | .060 |
|  | Based on Median and with adjusted df | 3.239 | 4 | 2.571 | .205 |
|  | Based on trimmed mean | 3.611 | 4 | 10 | .045 |

| **Multiple Comparisons** | | | | | | |
| --- | --- | --- | --- | --- | --- | --- |
| Dependent Variable: Frass | | | | | | |
| Games-Howell | | | | | | |
| (I) Storage time | (J) Storage time | Mean Difference (I-J) | Std. Error | Sig. | 95% Confidence Interval | |
|  |  |  |  |  | Lower Bound | Upper Bound |
| 0 month | 12 months | -.00733 | .00120 | .078 | -.0166 | .0019 |
|  | 24 months | -.03700 | .00902 | .160 | -.1064 | .0324 |
|  | 36 months | -.22167 | .13367 | .585 | -1.2502 | .8068 |
|  | 48 months | -3.08033^*^ | .39754 | .049 | -6.1390 | -.0216 |
| 12 months | 0 months | .00733 | .00120 | .078 | -.0019 | .0166 |
|  | 24 months | -.02967 | .00910 | .230 | -.0970 | .0376 |
|  | 36 months | -.21433 | .13368 | .605 | -1.2427 | .8140 |
|  | 48 months | -3.07300^*^ | .39754 | .050 | -6.1317 | -.0143 |
| 24 months | 0 months | .03700 | .00902 | .160 | -.0324 | .1064 |
|  | 12 months | .02967 | .00910 | .230 | -.0376 | .0970 |
|  | 36 months | -.18467 | .13398 | .690 | -1.2049 | .8355 |
|  | 48 months | -3.04333 | .39764 | .050 | -6.0992 | .0125 |
| 36 months | 0 month | .22167 | .13367 | .585 | -.8068 | 1.2502 |
|  | 12 months | .21433 | .13368 | .605 | -.8140 | 1.2427 |
|  | 24 months | .18467 | .13398 | .690 | -.8355 | 1.2049 |
|  | 48 months | -2.85867^*^ | .41941 | .041 | -5.4834 | -.2339 |
| 48 months | 0 month | 3.08033^*^ | .39754 | .049 | .0216 | 6.1390 |
|  | 12 months | 3.07300^*^ | .39754 | .050 | .0143 | 6.1317 |
|  | 24 months | 3.04333 | .39764 | .050 | -.0125 | 6.0992 |
|  | 36 months | 2.85867^*^ | .41941 | .041 | .2339 | 5.4834 |
| *. The mean difference is significant at the 0.05 level. | | | | | | |
